# Hippocampal Inactivation with TTX Impairs Long-Term Spatial Memory Retrieval and Modifies Brain Metabolic Activity

**DOI:** 10.1371/journal.pone.0064749

**Published:** 2013-05-28

**Authors:** Nélida María Conejo, José Manuel Cimadevilla, Héctor González-Pardo, Marta Méndez-Couz, Jorge Luis Arias

**Affiliations:** 1 Laboratory of Neuroscience, Department of Psychology, University of Oviedo, Oviedo, Spain; 2 Department of Neuroscience, University of Almeria, Almeria, Spain; Rosalind Franklin University, United States of America

## Abstract

Functional inactivation techniques enable studying the hippocampal involvement in each phase of spatial memory formation in the rat. In this study, we applied tetrodotoxin unilaterally or bilaterally into the dorsal hippocampus to evaluate the role of this brain structure in retrieval of memories acquired 28 days before in the Morris water maze. We combined hippocampal inactivation with the assessment of brain metabolism using cytochrome oxidase histochemistry. Several brain regions were considered, including the hippocampus and other related structures. Results showed that both unilateral and bilateral hippocampal inactivation impaired spatial memory retrieval. Hence, whereas subjects with bilateral hippocampal inactivation showed a circular swim pattern at the side walls of the pool, unilateral inactivation favoured swimming in the quadrants adjacent to the target one. Analysis of cytochrome oxidase activity disclosed regional differences according to the degree of hippocampal functional blockade. In comparison to control group, animals with bilateral inactivation showed increased CO activity in CA1 and CA3 areas of the hippocampus during retrieval, while the activity of the dentate gyrus substantially decreased. However, unilateral inactivated animals showed decreased CO activity in Ammon's horn and the dentate gyrus. This study demonstrated that retrieval recruits differentially the hippocampal subregions and the balance between them is altered with hippocampal functional lesions.

## Introduction

Solving the memory puzzle involves understanding the hippocampal role in spatial behaviour, clarifying the particular contribution of both hippocampi and its interaction with other brain structures. Several studies described the effect of unilateral hippocampal interventions on memory tasks. In this regard, it seems well established that partial hippocampal inactivation with tetrodotoxin (TTX) or lidocaine caused severe memory problems in different hippocampal-dependent tasks, like the Morris water maze, rotation arenas or passive avoidance tasks, by altering those processes engaged in memory formation [1], [2], [3], [4]. In the last years, it was shown that the hippocampus can be necessary for retrieving memories acquired days or weeks before [5], [6], [7]. However, the comparison of functional unilateral versus bilateral inactivation in long periods after acquisition has not been explored yet.

The assessment of brain activity during such experimental manipulations using cytochrome oxidase (CO) histochemistry has shed light on the interactions between the hippocampus and other related structures [8], [9], [10]. These results could provide evidence about the role of different brain structures engaged to any of the phases of memory formation during the learning experience. In this way, hippocampo-cortical functional integrated circuit seem relevant for successful performance and retrieval of spatial memory [11], [12], [13], [14], [15], [16].

CO is a mitochondrial enzyme that catalyzes the transfer of electrons to oxygen generating ATP via the coupled process of oxidative phosphorylation [17]. CO activity reflects changes in the brain metabolic capacity induced by energy requirements, and CO activity is regulated by and closely correlated with brain functional activity [18], [19].

Several authors demonstrated CO changes in memory circuits associated with spatial memory after several experimental manipulations. Hence, it was applied to discern how different structures modify their metabolic demands in subjects solving working memory tasks [20] or under other experimental manipulations [9], [21].

However, it is not clear how the hippocampal system and related structures functionally interact when the hippocampus is unilaterally or bilaterally inactive and the subject is forced to recall spatial information learned several weeks before with an intact brain. Similarly, it is unknown how hippocampal inactivation may affect the functional interrelationships between the hippocampus and prefrontal cortex, and therefore affect the spatial behavior. Here we applied CO histochemistry to determine the brain metabolism in rodents that have to retrieve long-term memories in the Morris water maze under unilateral or bilateral hippocampal reversible inactivation. In the same way, interregional CO activity correlations among medial prefrontal cortex and dorsal hippocampus are also used to determine functional changes in the neural networks therein following cerebral inactivation.

## Materials and Methods

### Animals

Thirty male adult Wistar rats (300–350 g) from the breeding colony of the University of Oviedo (Oviedo, Spain) were used in this study. They were housed under standard conditions (12-h light/dark cycle with lights on from 08:00–20:00h), at constant room temperature of 21±2 °C with ad libitum access to food and water. All experimental procedures carried out with animals were approved by a bioethics committee of the University of Oviedo and strictly followed the European Communities Council Directive (2010/63/UE) and the Spanish legislation (R.D. 1201/2005) for the care and use of experimental animals.

### Surgery

Rats were anesthetized with ketamine (100 mg/kg i.p.) and xylazine (5 mg/kg i.m.) and given additional doses of ketamine i.p. as needed to maintain deep anaesthesia. Subjects were placed in a stereotaxic frame (Narishinge, Tokio, Japan) and the scalp was incised and retracted. The skull was exposed and adjusted until bregma and lambda were on the same horizontal plane. After small burr holes were drilled, stainless-steel cannulae (26 gauge) were implanted bilaterally or unilaterally in the dorsal hippocampus (coordinates relative to bregma: AP –3.5 mm, ML ±2.5 mm, DV –2.00 mm from dura) according to Paxinos and Watson's Atlas [22]. Cannulae were secured using dental cement and anchoring screws.

### Apparatus

Animals were trained in the Morris water maze, using a circular water tank made of black fibreglass (diameter  = 1.5 m and height  = 75 cm) placed 50 cm above the floor [23]. The pool was filled with tap water to a height of 32 cm and a black escape platform was placed 2 cm beneath the water surface. The water temperature was kept at 23±1°C during the entire test period. The experimental room had numerous visual cues on the walls such as posters, plastic dishes, and a shelf. The swimming pool was indirectly illuminated by two halogen spotlights (500 W) located on the floor and facing the walls. The Morris water maze was divided virtually into four quadrants, according to the cardinal points (N, S, E, W) and swimming paths were recorded and analyzed using a computerized video-tracking system (Ethovision Pro, Noldus Information Technologies, Wageningen, The Netherlands).

### Behavioural procedure

#### Habituation

Rats were allowed to recover for 7 days during which they were handled daily. On day one, each rat received two habituation sessions spaced 1 h apart. Rats were randomly released four times per session, facing the pool wall from one of the four compass locations around the pool. Subjects were returned to their home cages between sessions. The escape platform used on the first day was painted white and stood up 2 cm above the water surface. Rats were allowed to freely swim to locate the escape platform or placed on it if 60s had elapsed. They remained on the platform for 15 s. Then they were introduced into a black plastic bucket for 30 s. The water was stirred between trials in order to remove olfactory traces of previous swim patterns [24].

#### Training phase

After the habituation phase, each animal received a single four-trial session during five consecutive days, days 2 to 6. The platform remained in the same position as during habituation. In each trial, the subjects were released randomly from one of four compass locations and had to search for a hidden platform that remained in the same position during the whole training period. On day 6, after completing the last trial of the training phase, each rat was subjected to a probe trial. The escape platform was removed and subjects were introduced during 30 s from the quadrant opposite to the target quadrant.

#### Intracerebral Injections

Tetrodotoxin (TTX), a highly selective voltage-gated sodium channel blocker, was used to temporally inactivate the dorsal hippocampus. Twenty-eight days after finishing the training rats received 1 µl of saline or 5 ng of TTX in 1 µl of saline. During infusions, rats were placed on the experimenter's lap, where grooming or excessive motion were limited. An injection cannula (32 G) protruding 2 mm from the guide cannula was inserted into the hippocampus. The injection solution was delivered during 90 s using a Hamilton syringe connected to the injection cannula with a short piece of polyethylene tubing. The injection cannula was left in place for an additional 1 min to achieve a proper diffusion of the drug from its tip. Tissue inactivation lasts approximately 3 h [25].

Subjects were randomly assigned to any of the three groups: bilateral TTX injections (BIL; n = 10), right unilateral TTX injections (RU; n = 10), and saline injections (CTR; n = 10). Rats were subsequently returned to their home cages, and any abnormalities in movement were examined for 30 min before they were placed into the maze for the remote memory probe.

#### Remote Memory Probe

The remote memory probe began 45 min after the intracerebral injection. Subjects were released from the quadrant opposite to the target quadrant and allowed to swim for 30 s. Time spent in each quadrant and total distance swum were recorded and analyzed later using the video-tracking system. Additionally, the pool was also conceptually divided into a central circular area and two concentric annular areas (inner, middle and outer areas, respectively). The total number of visits and swimming time in these rings were used to evaluate the exploratory activity of each group.

### Quantitative Cytochrome Oxidase Histochemistry

Ninety minutes after the behavioral procedures, rats were decapitated and their brains quickly frozen in isopentane. Coronal brain sections (30 µm thick) were obtained using a cryostat microtome (Microm HM-505E, Heidelberg, Germany) and processed for CO histochemistry according to the method described by Gonzalez-Lima and Jones [26]. A total of twelve measurements (four readings in three consecutive coronal sections) were taken per brain region. These measurements were averaged to obtain one mean value per region for each animal and were expressed as arbitrary units of optical density (OD). In order to quantify enzymatic activity and to control staining variability across different staining baths, slides including sets of tissue homogenate standards obtained from adult male Wistar rat brains were included in the study. These standards were cut at different thicknesses (10, 30, 40, and 60 µm) and included in each staining bath with the rest of slides. Previously, mean cytochrome oxidase (CO) activity of the homogenate was spectrophotometrically assessed. Therefore, sets of sections from rat brain homogenate of known CO activity were used as calibration standards in each CO staining bath. Series of coronal sections from each brain together with a complete set of standards were used to perform CO histochemistry.

Briefly, slides were lightly fixed for 5 min with a 1.5% glutaraldehyde, rinsed three times in phosphate buffer and preincubated in a solution containing cobalt chloride and dimethylsulfoxide dissolved in Tris buffer. Once the sections had been rinsed in phosphate buffer (pH 7.6; 0.1 M), they were incubated in darkness for 1 h at 37°C in a solution containing diaminobenzidine, sucrose, cytochrome c and catalase (Sigma-Aldrich, Spain) dissolved in phosphate buffer (pH 7.6; 0.1 M), which was continuously stirred. The slides were rinsed three times with cold phosphate buffer, and then dehydrated and coverslipped with Entellan (Merck, Darmstadt, Germany).

Regression curves between section thickness and known CO activity measured in each set of standards were calculated for each incubation bath. Finally, average regional optical density measured in each brain region was converted into CO activity units (micromoles of cytochrome c oxidized/min/g tissue wet weight at 23°C) using the calculated regression curve in each homogenate standard. CO histochemical staining intensity in each brain region of interest was measured densitometrically and converted to CO units using a computer-assisted image analysis workstation (MCID, InterFocus Imaging Ltd., Linton, England) composed of a high precision illuminator, a digital camera and a computer with specific image analysis software. CO activity in both the right and left hemispheres of the selected brain regions (located in the cortex, diencephalon and amygdala) were previously measured in every subject. However, no significant differences between right and left hemispheres were found. Therefore, we decided to show only these brain regions in the right hemisphere. Eight brain regions were measured unilaterally in each subject. In addition, the prefrontal cortex and dorsal hippocampus were measured bilaterally. The dorsal part of the hippocampus (CA1, CA3 and DG areas) was measured approximately between –4.30 and –4.40 mm anterioposterior from bregma (Paxinos & Watson’s rat brain atlas) in order to avoid possible direct effects of TTX diffusion from the injection site at –3.5 mm. The actual extension of the TTX area of influence at the injection site was estimated in previous pilot studies to be on average less than 1.5 mm in diameter.

Six animals, four from BIL group and two from RU group were discarded after the histology since cannulae did not reach the hippocampus. According to this, the final number of subjects per group was: CTR n = 10, RU n = 8, BIL n = 6.

### Statistical Analysis

#### Behavioural Data

Mean escape latencies during the training phase were analysed using two-way repeated measures ANOVA (group x days). Similarly, two-way repeated measures ANOVAs (group x quadrant) were used to evaluate differences between groups in mean time spent in the different quadrants during the retention and remote memory probes. In addition, the mean number of visits and time spent in the previously mentioned circular concentric areas in the remote memory probe were analyzed with two-way repeated-measures ANOVAs (group x area). Finally, the total distance swum during the remote memory probe was evaluated with one-way ANOVA. Tukey’s HSD post-hoc tests were applied when significant ANOVA results were found.

#### CO activity

Differences in CO activity between experimental groups in each brain region were evaluated by one-way ANOVA. Tukey’s post hoc tests were used when ANOVA indicated significant group differences. In order to evaluate possible changes in hippocampal functional connectivity caused by TTX injections, regional CO activity data were analyzed using pair-wise correlations between the hippocampal areas in each experimental group. The analysis of interregional correlations was done by calculating Pearson product-moment correlations. CO activity values were normalized by dividing the measured activity of each structure by the average CO activity value of the hippocampal areas measured for each animal. This was done to reduce variation in the intensity of the CO staining not resulting from experimental manipulation. In addition, in order to avoid errors derived from calculation of multiple correlations using small sample sizes we used a ‘jackknife’ procedure [27] based on the calculation of all possible pair-wise correlations resulting from removing one subject each time and taking into consideration only those correlations that remain significant (p<0.01) across all possible combinations. Statistical analysis was performed using statistical analysis software (SigmaStat 3.5, Systat Software, San Jose, California, USA).

## Results

### Behavioural Results

**Figure 1:**
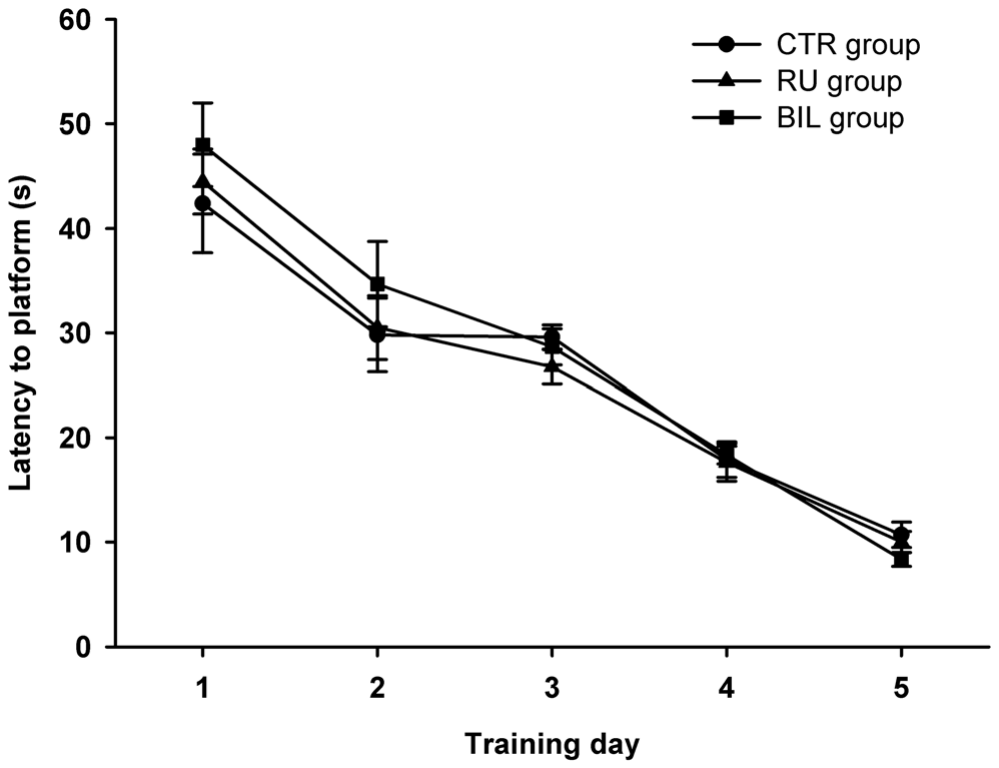
Learning Curves. Similar mean escape latencies across training days in the water maze of the three experimental groups. Data are presented as mean ± S.E.M.

**Figure 2:**
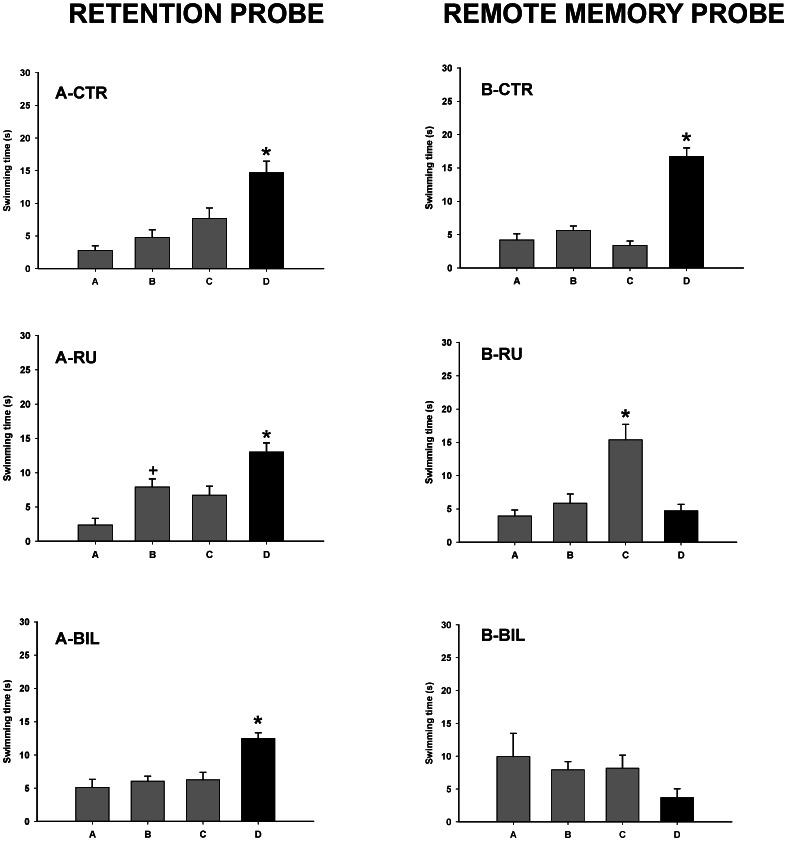
Retention probe and remote memory probe. Mean time spent in the different quadrants during retention probe (left column) and after TTX injection (remote memory probe, right column) in the different experimental groups. Bars represent mean swim latencies in the different quadrants of the water maze during the probes. D =  target quadrant, C =  opposite, A =  counter-clockwise, B =  clockwise. *p<0.01, significantly different as compared to the rest of quadrants, ^+^ *p*<0.05, significantly different as compared to quadrant A. CTR: control, RU: right, and BIL: bilateral groups.

Groups did not differ in their latency to find the hidden platform (*F*_2,21_ = 0.23; *p*>0.05) but there was a significant main effect of days (*F*_4,84_ = 74.9; *p*<0.001) and no interaction (*F*_8,84_ = 0.37; *p*>0.05). Tukey HSD test revealed that subjects learned the task, since latencies decreased significantly across sessions in the five training days (*p*<0.05) (Fig. 1). Additionally, groups did not differ during the retention probe (*F*_2,21_ = 1.57, *p*>0.05) but there was significant main effect of quadrant (*F*_3,63_ = 23.2; *p*<0.001). Post hoc analysis showed that subjects remembered the position of the hidden platform since they spent more time swimming into the target quadrant (*p*<0.01) (Fig. 2).

When subjects received saline or TTX unilaterally or bilaterally, the data analysis of the remote memory probe showed an interaction between group and quadrant (*F*_6,63_ = 13.1; *p*<0.001). Post hoc analysis revealed that CTR animals remembered the platform location twenty eight days later, spending more time swimming in the escape quadrant (*p*<0.001). However, RU and BIL groups did not search for the missing platform in the correct quadrant. Hence, RU group showed a significant trend to swim in quadrant C (*p*<0.001), whereas BIL group showed no preference for any quadrant (Fig. 2).

Analysis of the number of visits to the predefined concentric circular areas showed significant effects of group (*F*_2,21_ = 3.87; *p*<0.05) and circular area (*F*_2,42_ = 40.7; *p*<0.001), and no interaction (*F*_4,42_ = 0.9; *p* = 0.4). Post hoc test showed a strong tendency in RU and BIL groups to cross more frequently the limits of the rings than CTR group (p = 0.06). No significant group differences were found in the total distance swum (F_2, 21_ = *p*>0.05).

### Mean Brain CO Activity

Quantification of CO activity in the dorsal hippocampus showed differences between groups in CA1 area (right: *F*_2,21_ = 121.3; *p*<0.001 and left: *F*_2,21_ = 196.6; *p*<0.001) and CA3 area (right: *F*_2,21_ = 71.3; *p*<0.001 and left: *F*_2,21_ = 23.2; *p*<0.001). Post hoc analysis showed that BIL group had significantly higher CO activity in the CA1 and CA3 areas (*p*<0.001) in both hemispheres. Moreover, CO activity was higher in the CTR group compared to RU group in CA1 and CA3 areas of both hemispheres (*p*<0.01).

Regarding the dentate gyrus (DG), ANOVA disclosed significant differences between groups in the right DG (*F*_2,21_ = 36.7; *p*<0.001) and left DG (*F*_2,21_ = 13.8; *p*<0.001). In the right DG, CTR group showed higher CO activity compared to the other groups (p<0.05), and BIL group displayed higher CO activity as compared to RU (*p*<0.05). In the left DG, CTR and BIL groups exhibited higher CO activity than RU group. Mean regional CO activity measured in the experimental groups is summarized in Table 1. We found group differences in only cingulate area, with BIL group had higher CO activity in left hemisphere (*F*_2,21_ = 9.3; *p*<0.001). See Table 2.

**Table 1:** Mean CO activity measured in hippocampal regions.

	CTR	BIL	RU
**Left Hippocampus**			
CA1 area	31.6±1.0^+^	42.4±0.9*	13.7±0.8
CA3 area	30.5±1.7^+^	38.2±1.8*	18.6±2.2
Dentate gyrus	38.8±2.6	33.4±1.8	23.7±1.1*
**Right Hippocampus**			
CA1 area	29.6±1.1^+^	41.6±0.7*	15.8±1.2
CA3 area	31.8±1.0^+^	40.1±0.9*	18.6±1.3
Dentate gyrus	36.4±1.3^+^	31.7±0.8*	22.0±1.1

* *p*≤0.01, significantly different from the rest of groups (Tukey’s tests), ^+^ *p*≤0.01, significantly different as compared to RU group.

**Table 2:** Mean CO activity measured in prefrontal areas.

	CTR	BIL	RU
**Left Prefrontal Cortex**			
Prelimbic Area	26.2±1.1	25.1±0.8	23.8±0.9
Infralimbic Area	21.4±0.9	22.3±0.1	20.6±1.8
Cingulate Area	25.0±0.7	26.4±1.3	27.5±0.9
**Right Prefrontal Cortex**			
Prelimbic Area	24.5±0.6	25.0±1.2	21.6±0.6
Infralimbic Area	23.1±0.6	25.1±0.6	21.1±1.0
Cingulate Area	22.7±0.4	26.8±0.7*	22.2±0.9

* *p*≤0.01, significantly different from the rest of groups (Tukey’s tests).

As regards to the rest of brain regions quantified, group differences emerged in the lateral mammillary nucleus and the entorhinal cortex (*F*_2,21_ = 17.7; *p*<0.001 and *F*_2,21_ = 27.2; *p*<0.001 respectively). Post hoc test showed higher CO activity levels in all experimental groups (RU and BIL) as compared with the CTR group (*p*<0.05). Activity differences also appeared in the dorsal thalamic nucleus (*F*_2,21_ = 7.7; *p*<0.01), the perirhinal cortex (*F*_2,21_ = 26.7; *p*<0.001) and the basolateral amygdala (*F*_2,21_ = 6.44; *p*<0.01). Post hoc test revealed that BIL group had higher CO activity as compared to the rest of groups in all those regions (*p*<0.05). See Table 3 for additional brain regions quantified.

**Table 3:** Mean CO activity (±S.E.M.) measured in selected brain regions of the different experimental groups.

	CTR	BIL	RU
**Cortex**			
Entorhinal	14.4±0.3*	19.9±0.8	18.5±0.6
Perirhinal	13.1±0.3	18.1±0.4*	14.4±0.6
**Diencephalon**			
Anterodorsal thalamic nucleus	30.6±0.5	34.1±0.5*	31.9±0.7
Anteroventral thalamic nucleus	24.6±0.4	21.6±0.8	23.2±1.0
Medial mammillary nucleus	30.2±0.5	31.1±1.2	30.5±1.1
Lateral mammillary nucleus	27.4±0.6*	31.5±0.4	31.2±0.4
**Amygdala**			
Lateral nucleus	14.8±0.6	16.6±0.8	14.6±0.8
Basolateral nucleus	21.3±1.1	25.9±0.1*	20.7±0.7

* *p*≤0.01, ^+^ *p*≤0.05 significantly different from the rest of groups (Tukey’s tests).

### Interregional within-group correlations of hippocampal CO activity

**Figure 3:**
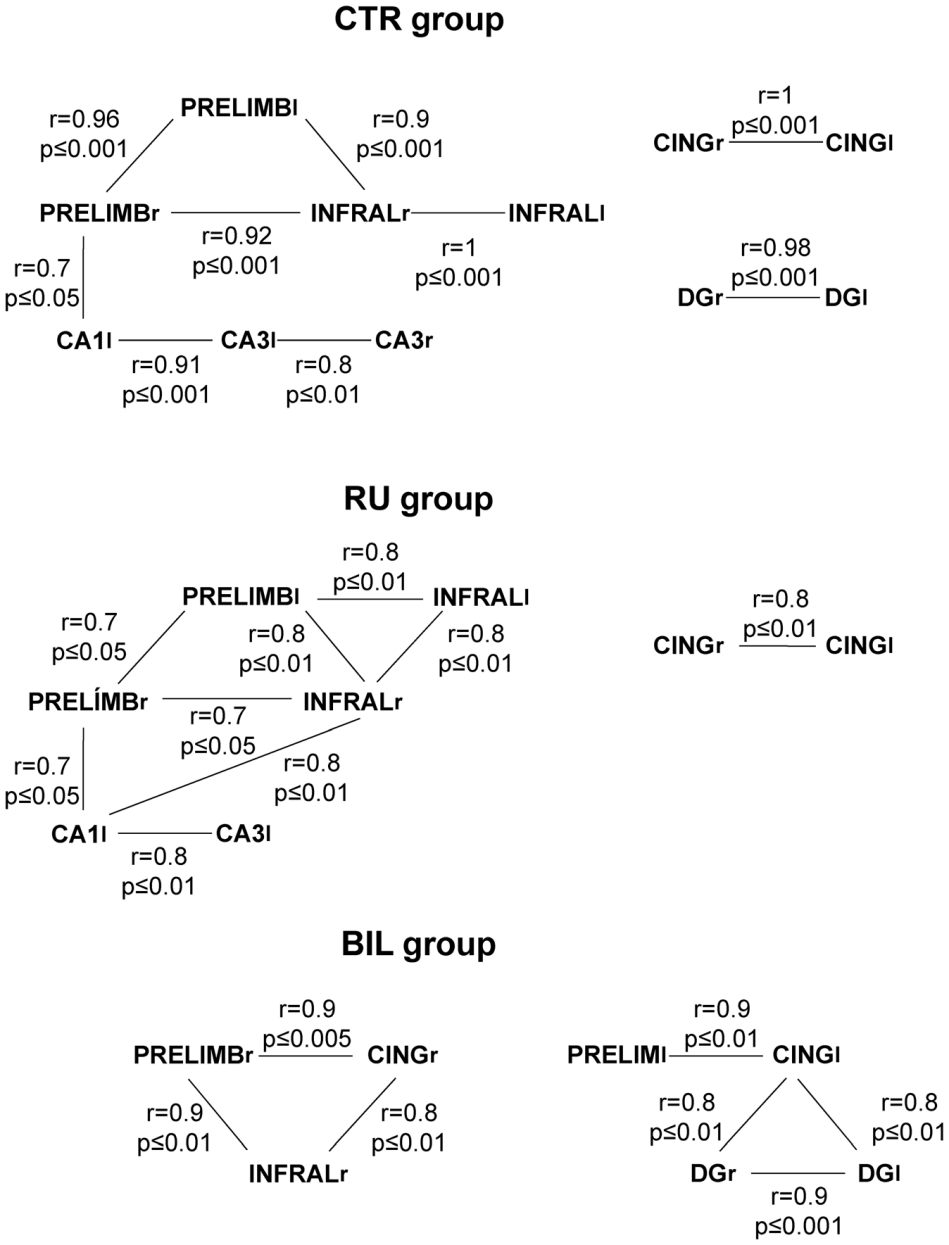
Interregional within-group correlations of CO activity. Schematic diagram showing significant correlations in CO activity among right (R) or left (l) between hippocampal and prefrontal regions calculated for the different experimental groups. Abbreviations: prelimbic (PRL) and infralimbic (IL) cortex, cingulate cortex (CG), hippocampal dentate gyrus (DG) and subfields (CA1 and CA3).

Significant regional correlations were found in particular areas of the right and left hippocampus in the different experimental groups (Fig. 3). A negative cross-correlation between the right CA1 area and the right DG was found in the CTR group. The BIL group showed positive correlations among the left and right DG and the right CA3 area. However, the RU group had significant correlations limited to the left hippocampus (Fig. 3).

## Discussion

### Unilateral Inactivation Impaired Retrieval as Much as Bilateral Inactivation

This study showed that dorsal unilateral and bilateral hippocampal inactivation has similar effects on retrieval of memories acquired 4 weeks before. The time period used to evaluate remote memory was based on previous studies using one month (28 days) to evaluate long-term or remote memory after hippocampal inactivation or lesion [28], [29], [30]. Both treatments impaired performance in the remote probe test in the Morris water maze. Subjects did not remember the position of the hidden platform. This result agrees with previous works reporting the hippocampal involvement in retrieval of spatial memories acquired several weeks before in the Morris water maze [5], [6], [7]. Therefore, our results agree with recent evidence about hippocampal recruitment during spatial memory retrieval [30].

Despite the disturbance of spatial memory in both groups, it is noteworthy that the unilateral and bilateral inactivation altered spatial memory in a different way. Hence, whereas bilateral treatment subjects distributed the searching around the pool, unilateral inactivated subjects showed a marked preference for the lateral quadrant. This probably shows that unilateral treated subjects preserve some memories although inaccurate about the goal, similarly to the alterations manifested by rats that received hippocampal inactivation after training, knowing how but not where [31].

The effects of unilateral hippocampal inactivation on behaviour are to some extent controversial. Unilateral blockades not always impair hippocampal-dependent behaviours. In order to understand this effect we probably need to pay attention both to the task used and the memory phase affected by the treatment. Therefore, in very spatial-demanding tasks like the Morris water maze or active place avoidance arenas, unilateral inactivation alters all phases of memory formation, as shown by different studies carried out during the last 20 years [1], [3], [32]. However, the same interventions do not consistently alter memories in hippocampal-dependent tasks where orientation demands are low. This is the case of passive avoidance tasks, where orientation and navigation in this environment in limited and demands are more related to context recognition [4], [8], [33].

On the other hand, it is necessary to consider the memory phase interrupted during hippocampal inactivation. Retrieval was demonstrated to be more prone to interference than other phases of memory formation. As Moser and Moser [2] showed, the amount of hippocampal tissue required for retrieval is higher than that needed for acquisition.

Other phases of memory formation were also tested under unilateral and bilateral hippocampal interventions and similar results were found. Hence, when intrahippocampal injections of TTX were applied to block consolidation, unilateral and bilateral treatments did not differ [34]. So, although unilateral blockade theoretically leaves the contralateral hippocampus intact to hold a memory, one hippocampus cannot be enough to support and adequately process spatial memories. We have to consider that cognitive alterations after unilateral blockade could be caused by a plausible interference between the inactivated and untreated hippocampi. In this respect, it is well known that each hippocampus sends and receives fibres from the contralateral hippocampus [35], and unilateral lesion of one hippocampus can disturb physiological processes in the contralateral side [36], [37].

It is also possible that the spatial memory was lateralized to the right hippocampus [38], and as a consequence of this, right hippocampal inactivation impaired spatial memory retrieval. However, this point is not clear. Right and left hippocampal inactivation showed, in fact, subtle behavioural effects [38] while other authors did not detect them [1]. Moreover, the role of each hippocampus in spatial behaviour is also matter of debate in humans. Hence, unilateral epileptic focus in the medial temporal lobe or unilateral hippocampal removal is enough to prevent spatial learning in virtual reality tasks, and this can be independent of the side of the brain involved [39], [40].

### Hippocampal Blockade Modifies Metabolic Activity in Several Structures Involved in Spatial Orientation

Cytochrome oxidase histochemistry (CO) was used to assess brain energy metabolism of several brain structures that could be involved in the solution of this task. Previous works showed that CO activity can reflect metabolic changes linked to learning and memory processes [8], [10], [21], [41].

Our study proved that DG, CA3 and CA1 manifested different metabolic activity according to the treatment received. CTR group displayed positive correlations between right and left DG areas and between ipsilateral CA areas. Also contralateral CA3 areas showed positive correlations between them. This pattern is altered as the hippocampal activity is blocked. DG and CA3 regions were proposed to process the geometry of the environment [42], being essential mossy fibre inputs to CA3 for encoding spatial information [43]. Furthermore, unlike the other groups, bilaterally inactivated animals showed dissociation regarding CO activity found in different regions of the hippocampus. The animals with bilateral inactivation showed increased activity in CA1 and CA3 areas during retrieval, while the CO activity of the dentate gyrus largely decreased. It may be that CA1/CA3 areas and the dentate gyrus have opposing functions during different phases of spatial memory processing. Some authors [44], [45] have demonstrated that the perforant path input to CA3 area is critical for memory retrieval processes (related to a pattern completion mechanism) whereas the dentate gyrus is critical for memory encoding processes (as probably related to spatial pattern separation mechanisms). This means that impaired learning or general memory deficits found in an animal never being able to perform a task are not indicative of impaired pattern completion [46]. The different CO activity observed between Ammon’s horn areas and the dentate gyrus may be indicative of this dissociation, since during memory retrieval, spatial pattern completion is essential in order to recover the full stored information, but pattern separation, which occurs at the time of encoding and storage, is not essential, and for this reason the dentate gyrus appears to be inhibited during expression/retrieval.

Since the hippocampus is needed for an adequate orientation, partial bilateral and unilateral inactivation caused alterations in other structures that develop an important role in the orientation system of the brain. Hence, patterns of correlations slightly changes in RU group and is very altered in BIL group. This loss of positive correlations supports the hypothesis that TTX impaired the network involved in retrieval of spatial memories. Note that the comparison of different correlations between hippocampal components provides information about the neural net that underlies the behavioural processes studied. In this regard, it was demonstrated that analyses at the level of neural networks were more sensitive to understand brain dysfunctions than attending only to the parts that integrate the system [47].

We also did pay attention to the changes of metabolic activity in the groups of study. Our work showed that an impaired behavioural performance did match with an increase of the brain activity in the entorhinal cortex and lateral mammillary nucleus revealed by CO histochemistry. CTR group showed reduced CO activity in the entorhinal cortex in comparison with all treated groups. It is well known that the entorhinal cortex is profusely connected with the hippocampal system and contains cells which are suggested to be specialized in the coding of spatial information [48]. Moreover, lesions of the dorsolateral area of the entorhinal cortex were reported to impair retrieval of spatial memories acquired one week before [49]. Since the hippocampal system physiology is disrupted by TTX injections, this could trigger an increase in the activity of those brain structures involved in retrieval of memories. An alternative hypothesis suggests that unsuccessful attempts of finding out the position of the platform would increase the exploratory activity and the CO metabolism in the entorhinal cortex. As shown before, exploratory activity can regulate the activity of the entorhinal cortex. Matrov et al. [50] reported that the rats that displayed high rates of exploratory activity increased their oxidative metabolism in the entorhinal cortex. As we described with respect to the frequency of visiting the different ring-segments of the MWM, inactivated groups changed segment more frequently than controls, although no differences were found in the total distance covered.

Similar metabolic patterns were displayed in other brain regions involved in spatial orientation. The lateral mammillary bodies and anterodorsal thalamic nucleus are known to take part of the Papez circuit and the head direction system [51] which contributes to the processing of both allocentric and geometric cues [52]. Moreover, the lateral mammillary nucleus directly projects to the anterodorsal thalamic nucleus via the mammillothalamic tract [53]. Accordingly, lesions of the mammillothalamic tract impair allocentric and egocentric spatial navigation in the water maze [54]. Previous studies demonstrated that CO activity changes in the lateral mammillary bodies after learning in a spatial working memory task [20], [55]. In our work, BIL and RU groups showed an increased activity when compared with the CTR group. Regarding the anterodorsal thalamic nucleus, we found a higher CO activity in BIL group in comparison with the CTR group. Although the anterodorsal thalamic nucleus receives a major projection from the subiculum, the main output of the hippocampus, hippocampal lesions was reported not to disrupt head direction cell signals [56]. However, it is well known that the above-mentioned structures are part of the Papez circuit and during learning and memory processes these regions interact changing their metabolism [8]. So it would not be unusual that hippocampal inactivation produced changes in CO activity in these linked structures.

It is also necessary to point out that the BIL group increased its CO activity in many other several brain regions related to memory circuits. Hence bilaterally inactivated subjects increased CO activity in the perirhinal cortex, a brain structure that has been related to object recognition [57], [58] and discrimination [59], as well as spatial memory retrieval [60]. As Ramos [60] demonstrated, rats with perirhinal inactivation were impaired in retrieving spatial memories that were well acquired before the intervention. The activity in the cingulate cortex is also increased in BIL in comparison with CTR and RU groups. This brain structure links cortical and limbic structures and it was reported to be involved in spatial memory in rats [61], [62]. Finally, other structures like prelimbic and infralimbic cortices did not reflect any change in their CO activity, and probably shows that they were not directly involved or detected by CO histochemistry after the retrieval of spatial information required in our experiment. As reported before, infralimbic and prelimbic cortices are important in attentional processes and flexibility of behaviour [63] but they are also involved in memory extinction or consolidation of fear memories [64] that perhaps were not engaged in the retrieval phase of our spatial memory task. In agreement with our results, a recent study of remote spatial memory retrieval using both functional inactivation techniques and c-fos expression confirmed that only the cingulate cortex and not the prelimbic or infralimbic cortices is required for remote memory retrieval [30].

In conclusion, this experiment showed that retrieval of spatial memories depends on the integrity of the hippocampal system even several weeks after the initial training. However, since hippocampal inactivation altered metabolic activity in regions functionally related with the hippocampus, other regions could underlie the behavioural deficits registered. Moreover, inactivation of one hippocampus causes the same effect as bilateral blockade of this brain structure, an effect that has been reported in other hippocampal-dependent tasks [3].
